# Discovery of the Liver Hyaluronan Receptor for Endocytosis (HARE) and Its Progressive Emergence as the Multi-Ligand Scavenger Receptor Stabilin-2

**DOI:** 10.3390/biom9090454

**Published:** 2019-09-06

**Authors:** Paul H. Weigel

**Affiliations:** Department of Biochemistry & Molecular Biology, University of Oklahoma Health Sciences Center, Oklahoma City, OK 73104, USA; paul-weigel@ouhsc.edu; Tel.: +1-405-271-1288

**Keywords:** protein isoform, binding site, coated pit, glycosaminoglycan, endocytosis, phagocytosis, receptor recycling, systemic clearance

## Abstract

Since the discovery of a novel liver hyaluronan (HA) clearance receptor in 1981 by Laurent, Fraser and coworkers, 22 different ligands cleared by the renamed receptor (the Hyaluronan Receptor for Endocytosis (HARE); Stabilin-2 (Stab2)) were discovered over 37 years. Ligands fall into three groups: (1) 11 anionic polymers, (2) seven cleaved or modified proteins and (3) four types of cells. Seven synthetic ligands, not found normally in serum or tissues, likely mimic natural molecules cleared by the receptor. In 2002 we purified and cloned HARE, based on HA-binding activity, and two other groups cloned full-length receptor; FEEL-2 and Stab2. Macrophages likely require full-length Stab2 for efficient binding and phagocytosis of bacteria or apoptotic cells, since cell-binding domains are throughout the receptor. In contrast, all 16 known single-molecule binding sites are only within the C-terminal half (190HARE). The HARE isoform is generated by proteolysis, not mRNA splicing. The majority of circulating ligands is cleared by HARE, since sinusoidal endothelial cells of liver, spleen and lymph node express twice as many HARE half-receptors as full-length receptors. Based on their significant binding and functional differences, a modified receptor nomenclature is proposed that designates HARE as the C-terminal half-receptor isoform and Stab2 as the full-length receptor isoform.

## 1. The Beginning: Clearance of Hyaluronan, the First Ligand Discovered

In a 1997 interview [1], Swedish scientist Torvard Laurent described the simple, unpretentious birth of what would eventually become the story of the hyaluronan receptor for endocytosis (HARE) and Stabilin-2 (Stab2): “*My wife showed in Australia that there were small amounts (of hyaluronan) in blood and that it came via the lymph from the peripheral tissues. Bob Fraser then injected radioactive hyaluronan into rabbits to see how fast it disappeared from blood; he found that it disappeared with a half-life of 2.5 minutes. It went to the liver. So we started a large project to study the turnover of the polysaccharide in the organism.*” Thus, in exploratory experiments to assess what happens to circulating hyaluronan (HA^1^) in the vascular system of rabbits, Fraser et al. [2] discovered in 1981 that this polysaccharide rapidly accumulates in the liver. Laurent was an expert in the biophysical and chemical properties of HA and Robert Fraser was an expert in mammalian physiology and anatomy. Their collaboration provided an excellent combination of disciplines, perspectives and experimental techniques to perform a broad range of thorough and often elegant in vivo and in vitro studies over the next five years to characterize this systemic HA clearance activity. Table 1 lists the 22 known ligands [2,3,4,5,6,7,8,9,10,11,12,13,14,15,16,17,18] in their order of discovery over the 38 years since this seminal 1981 paper.

Fraser et al. [19] used whole body autoradiography to show that the liver was the organ responsible for the major amount of ^3^H-HA clearance in mice. Among the subsequently isolated specific liver cell types, Eriksson et al. [20] found that almost all the cleared HA was internalized and degraded by sinusoidal endothelial cells (SECs). These findings in live animals were then confirmed in vitro by Smedsrod et al. [21] in studies of the uptake activity in isolated liver cell types; only the SECs robustly endocytosed and degraded HA. In 1986, Laurent et al. [6] performed the first molecular characterization of HA binding to this newly identified clearance receptor. Cultured rat liver SECs bound either to large (6.4 MDa) ^3^H-HA with extremely high affinity, a K_d_ of 9 × 10^−12^ M, or to smaller ^3^H-HA (1.6 KDa) with a K_d_ of 4.6 × 10^−8^ M, which is still moderately high affinity. They estimated 10^5^ HA receptors per SEC.

The authors reasoned that due to the multi-valency of HA, multiple receptors could be engaged per HA molecule as a function of polymer length and thus, increasingly stronger binding constants might occur as HA size increases. Conversely, the number of HA molecules bound per cell would then decrease as the HA size (mass) increased. In this same 1986 study, Laurent et al. [6] reported that the HA clearance receptor also recognized chondroitin sulfate-A (CS-A), another member of the glycosaminoglycan (GAG) family of polysaccharides comprised of repeating disaccharide units with an uronic acid and an amino sugar (as for HA). Based on the ability of CS-A and HA each to inhibit binding of the other ligand, they concluded that the receptor binding sites for both ligands must overlap.

## 2. Naming the New Hyaluronan Clearance Activity—Why Not?

In hindsight, it seems very unusual that the two scientists did not name the activity responsible for the HA clearance they discovered and characterized in these ground-breaking studies. Although they clearly understood it to be a systemic endocytic (i.e., clearance) HA receptor, they did not formally propose a name for this activity in the titles, abstracts or discussions of their publications. I first met both Professors Fraser and Laurent in 1988 at a Ciba Foundation meeting on *The Biology of Hyaluronan* [22] and spoke with them many more times at meetings over several decades. Despite not naming the receptor in publications, they referred to it by its function calling it the “HA clearance receptor” in conversations and in presentations at conferences. Since this functional name is straightforward in meaning, most other scientists studying HA biology in a range of disciplines also called it the liver HA Clearance Receptor. In hindsight, I believe the following points may be reasons that Fraser and Laurent did not formalize a name for the receptor they discovered.

In the early1980s, before the information-explosions of molecular cloning and genome sequencing and the associated nomenclature surge, many investigators were studying HA receptors in different tissues and species. For example, that decade CD44 had been studied by other groups that named the same protein Pgp-1, In(Lu)-related p80, Hermes, ECM-III and HUTCH-I [23]. In the HA field, those studying HA binding proteins often used generalized names such as hyaluronectin or HA Binding Protein. It was an exciting, but confusing, period with dozens of groups studying HA binding activities using their own assays, in their species, tissues and cells of interest. Perhaps Laurent and Fraser chose not to name the HA clearance activity they discovered in a novel in vivo context because it might be an HA binding protein or receptor already named by others.

Both scientists were cautious with an inherently conservative approach regarding conclusions and nomenclature; they may have felt it was not appropriate for them to name the newly discovered HA clearance activity.

Although they were both extremely well trained and expert in their disciplines, neither Fraser nor Laurent were cell biologists, protein biochemists or molecular biologists. So, after discovering and characterizing this novel HA clearance activity over an exciting 5-year period, their focus shifted to resume the physiologic characterizations of systemic HA clearance in healthy animals and humans and in a wide range of disease, stressed or rare genetic conditions [24]. Neither of them studied the specificity or molecular characteristics of the new clearance receptor itself any further for the rest of their careers.

The HA clearance receptor would later be recognized as a broadly functional scavenger receptor, and it would be rediscovered and renamed several times in the context of discovering multiple different ligands. Sixteen years after its discovery, in a review focused on HA function and turnover [25], Torvard and Ulla Laurent and Robert Fraser still referred to the receptor they discovered as the “metabolic receptor for HA”.

## 3. Six Additional Ligands Are Discovered During the Pre-Cloning Era

Since the HA clearance receptor was discovered in 1981, 21 additional ligands have been identified (Table 1), most recently in 2018. Six new ligands were identified prior to receptor purification and cloning by several groups. In 1984 Blomhoff et al. [3] and Blomhoff et al. [4] showed, respectively, that acetylated-low density lipoprotein (AcLDL) and formaldehyde-treated albumin (i.e., formylated-albumin (fA)), are rapidly taken up by liver in live rats or in isolated liver SECs. Eskild et al. [5], proposed in 1986 that isolated rat liver SECs internalized fA by the same receptor that recognized CS-A, which was shown that year also to bind to the HA clearance receptor [6].

Smedsrod [7] discovered in 1988 that the free (cleaved) amino-terminal propeptide of procollagen III (PIIINP) was rapidly taken up by SECs and that HA and CS-A did not compete for (inhibit) this binding. Therefore, there was no evidence then that PIIINP uptake was mediated by the HA receptor. Six years later, Melkko et al. [8] reported that uptake of the ^125^I-N-terminal collagen I propeptide (PINP) was mediated by a SEC scavenger receptor that was not the HA/CS-A or mannose receptor. PINP and PIIINP inhibited the uptake of radiolabeled PINP by >95%, indicating that they both bind within the same site or to very close sites so that ligand bound to one site prevents or sterically hinders binding to the other proximal site. In contrast, fA, AcLDL and polyinosinic acid (pIA) partially inhibited endocytosis by 40%, 50% and 74%, respectively. Low density lipoprotein, HA and heparin (Hep) did not inhibit PINP endocytosis by SECs. The Smedsrod group concluded that PINP, PIIINP, fA, AcLDL and pIA are not recognized by the HA clearance receptor. At that time the possibility that multiple ligands could bind to discrete nonoverlapping sites in the same receptor protein may not have been widely appreciated or considered. Nonetheless, I conditionally include these molecules as HARE and Stab2 ligands, based on supporting results from several studies (described below) using recombinant receptor. Thus, seven ligand types were identified within 13 years after discovery of the new receptor. However, only four of these ligands (HA, CS-A, PINP and PIIINP are actually physiologic. AcLDL and fA, produced synthetically, are considered model compounds for naturally occurring variants of these proteins and although pIA does not occur naturally, it likely mimics GAG ligands.

## 4. More Ligands Are Discovered through 2018

**Figure 1:**
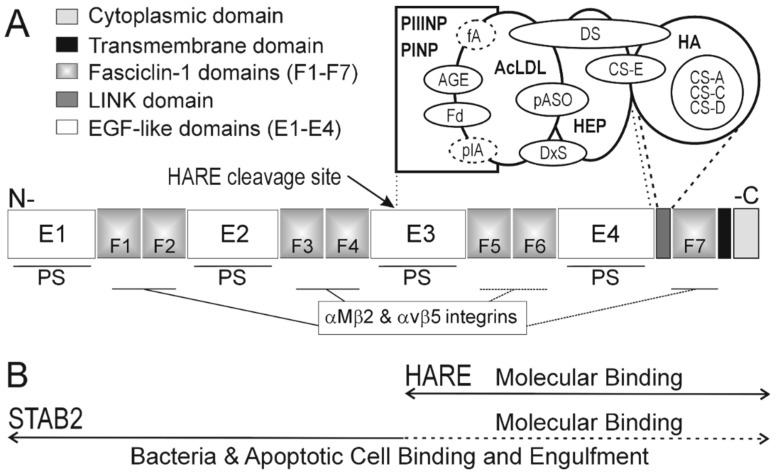
Organization of 19 ligand binding sites within domains of the two receptor isoforms, HARE and Stabilin-2 (Stab2). (**A**) The overall protein structure, with N-terminus left and C-terminus right, is schematically shown with different gray shadings indicating the main domains: cytoplasmic, transmembrane, Fasciclin-1 (F1–F7), LINK and Epidermal Growth Factor-like (E1–E4). The arrow indicates the proteolytic cleavage site that generates 190 kDa HARE (starting at Ser^1136^) from Stab2. Cleavage gives two approximate half-receptors: a soluble N-terminal end and a membrane bound C-terminal end. This C-terminal half-receptor is HARE, the functional hyaluronan (HA) receptor for endocytosis. The relative organization of the binding sites for 16 soluble molecular ligands are shown within the 190HARE region. Binding sites for the three cell-associated ligands, phosphatidylserine (PS) and αMβ2 and αvβ5 integrin, are indicated within the E1–E4 and F1–F7 domains, respectively, within the full-length protein. Although not yet identified, the bacterial binding sites are most likely within the E1–E4 and/or F1–F7 domains spanning most of the full-length receptor. A dashed line indicates ligands (fA and pIA) for which direct binding data using labeled ligands are not available. Overlapping symbols mean that the ligands mutually inhibit binding. (**B**) Based on all the known soluble ligand binding sites being within the C-terminal half of Stab2, this HARE isoform is designated as a molecular receptor (Section 10 and Section 11), whose function is to endocytose and degrade the 16 molecules identified as ligands so far. There is no evidence that any binding sites for these, or other, molecular ligands are present within the N-terminal half of Stab2. In contrast, intact Stab2 protein is designated as the receptor isoform large enough to mediate the efficient binding and engulfment of cells targeted by interactions along the whole protein between the E1–E4 and F1–F7 binding sites and target cell surface PS patches or integrins, respectively.

After the HA clearance receptor was purified and molecularly cloned, new research tools became available, including protein-specific antibody (Ab) and cell lines expressing recombinant proteins, and the nomenclature changed to HARE and then Stabilin-2 (Section 8 and Section 9). In this new era, many additional ligands were identified. Adachi and Tsujimoto [9] showed in 2002 that the receptor could bind both Gram–Negative and Gram–Postive bacteria, allowing macrophages to bind, phagocytose and kill these cells. The next year Tamura et al. [10] used recombinant protein cloned from human umbilical cord endothelial cells to identify advanced glycation end (AGE) products as receptor ligands. Interactions of AGE-receptors enable cells to endocytose and degrade proteins that have had free amine groups modified by an irreversible reaction with glucose. The browning or Mailliard reaction between protein amine groups and free glucose results in stable modification of lysine epsilon-NH_2_ groups to create stable Amadori rearrangement products [26]. AGE–product binding to cells expressing Stab2 was inhibited by AcLDL and pIA [10], indicating that the binding regions for these three ligands overlap (Figure 1A). Tamura et al. [10] also identified two more ligands by their ability to block AGE–product binding: fucoidan (Fd) and dextran sulfate (DxS). Fucoidan is a complex polysaccharide derived from a brown seaweed, which contains multiple components including fucose and sulfate. DxS is made by chemically sulfating dextran, which is a noncharged branched glucose polysaccharide.

In 2007, Jung et al. [11] reported that αMβ2 integrin is also a ligand and that lymphocytes expressing this cell surface protein can bind to specialized SECs expressing HARE and Stab2. Integrin can bind to each of the seven Fasciclin-1 (F) domains throughout the full-length Stab2 (F1–F7 in Figure 1A).

Six more ligands were found in 2008. Park et al. [12] identified phosphatidylserine (PS) as a HARE and Stab2 ligand, showing that this scavenger receptor enables macrophages to bind and engulf apoptotic lymphocytes with large areas of PS (patches) on their surface. Park et al. [27] then demonstrated that the four EGF-like domain repeats (E) in Stab2 (E1–E4 regions; Figure 1A) specifically bind PS and that Ca^+2^ is required for human monocyte-derived macrophages to phagocytose apoptotic cells by binding to PS on their surfaces. The E1–E3 regions contain six, whereas the E4 region has five, typical or atypical EGF-like domains. All four E1–E4 regions are able to bind PS and participate in engulfment of apoptotic cells. Kim et al. [28] also identified a conserved His residue within each of the E1–E4 regions that is proximal to a proposed PS binding loop and critical for activation of Stab2-mediated phagocytosis in the low pH (e.g., pH 6.8) environment associated with the inflammation and clearance of apoptotic cells. Also in 2008, Harris et al. [13] discovered that Hep clearance by liver SECs occurs via HARE and Stab2. Rat and human receptors specifically bind to small or large Hep [29], demonstrating that renal clearance is not the only mechanism for eliminating Hep from the circulation. Oie et al. [30] also reported that year that isolated liver SECs internalized Hep. However, since uptake was not inhibited by HA, they concluded that Hep does not bind to the HA receptor. In hindsight, a more appropriate conclusion would be that if Hep does bind to the HA receptor, it could not be within, or block, the HA binding site (as in Figure 1A). Finally, in 2008 Harris and Weigel [14] found that four other GAGs are ligands for the HA Clearance Receptor; CS-C, CS-D, CS-E and dermatan sulfate (DS). So, remarkably, six new ligands were discovered in just one year.

In 2011, a novel peptide ligand was reported by Lee et al. [15]. This group found that Stab2 is expressed in atherosclerotic plaques, present in macrophages as expected, but also in endothelial and smooth muscle cells within plaques. Using phage display and selection by binding to Stab2, the most prevalent specifically bound peptide was CRTLTVRKC. A basic local alignment search tool (BLAST) (by this author) identified no human proteins with this sequence; all top matches are microbial proteins. Although perhaps not physiologic (as with AcLDL, DxS, fA, Fd and pIA), this peptide behaved as a strong ligand. Peptide conjugated to chitosan nanoparticles or a fluorescent tag was targeted to atherosclerotic plaques in mice and co-localized with endothelial cells, macrophages, and smooth muscle cells. Kim et al. [16] reported in 2012 that a second integrin, αvβ5, enables Stab2-mediated engulfment of apoptotic cells (presenting surface PS patches) by forming a complex with the extracellular and/or membrane domains of Stab2.

More recently in 2016, Miller et al. [17] showed that HARE and Stab2 recognize and internalize phosphorothioate-modified antisense oligonucleotides (pASOs), which are used extensively as pharmacological and therapeutic agents. Gaus et al. [31] then determined that the backbone structure of these modified oligonucleotides is responsible for binding to HARE and Stab2. Most recently, in 2018, Swystun et al. [18] found that Stab2 mediates the turnover of von Willebrand Factor (VWF) in complex with Factor VIII (VWF•FVIII) in the circulation and thus regulates the half-life of these two clotting factors. Mature human VWF (2050 aa) is produced after cleavage of a signal peptide and a large (741 aa) N-terminal VWF propeptide [32].

## 5. Stabilin-2 Ligands Have Net Negative Charge and Fall into Three Groups

The 22 known ligands fall into one of three groups (Table 2): (1) anionic polymers, (2) proteins, and (3) cells. Seven GAGs, two polysaccharides and two nucleic acids comprise the first group of 11 polymeric ligands. The second group of seven protein ligands includes three normal unmodified, although cleaved, proteins (VWF, procollagen type I propeptide and procollagen type III propeptide) and three types of structurally altered proteins with modified amine groups; acetylated, glycated or formylated. Since the unmodified normal proteins are not ligands, it is the modified structures on former free amino groups that are recognized. Also included in this protein ligand group is the novel synthetic peptide identified by the ability to bind strongly to Stab2 within sclerotic plaques. The third ligand group includes four types of cells with specific surface characteristics: Gram–Negative and Gram–Positive bacteria (with as yet unknown surface ligands) and apoptotic cells (e.g., dying red blood cells or lymphocytes) expressing either surface αMβ2 or αvβ5 integrin and high surface PS levels (resulting in abnormally large cell surface PS clusters or patches).

A common characteristic among the three ligand groups is that all, except the synthetic peptide [15], have a net negative charge. All 11 anionic polymeric ligands are very high-density anionic polymers. Modified proteins are all more anionic than the normal proteins, due to the net loss of cationic amine groups. The integrins αMβ2 and αvβ5, PS, VWF, the N-terminal propeptides of procollagen types I and III, and bacterial cell surfaces are also all negatively charged at pH 7.

However, negative charge alone is not sufficient for binding to HARE or Stab2, indicating that negative charge patterns rather than just net charge may be recognized. For example, DNA and RNA are not bound effectively [33] and recombinant HARE or Stab2 do not bind to the anionic (but not sulfated) GAGs chondroitin or heparosan [14]. It is not known if the C-terminal half of full-length Stab2 can bind all the soluble ligands bound by the half-receptor HARE, with the same affinity and specificity (see Section 6, Section 10 and Section 11).

Of the 21 macromolecular or cellular ligands discovered thus far, six are synthetic or ceuticals and not found physiologically in humans: AcLDL, DxS, fA, Fd, pASO and pIA. Turnover and metabolism of the pASO ligand class of therapeutic drugs is certainly relevant physiologically as may be the metabolism of ingested Fd, which is marketed as a nutraceutical and may have anti-inflammatory, anti-cancer and other pharmaco-activities [34]. The DxS is very likely a mimic for Hep, since it completely blocks Hep binding [14]. Although pIA does not occur naturally, it likely mimics GAG ligands. The natural ligands mimicked by fA and AcLDL are unknown, but they are most likely AGE-derivatives of circulating albumin and LDL with naturally glycated amine groups (as occurs in diabetic patients).

## 6. Distribution of Ligand Binding Sites within the Two Receptor Isoforms

It was fortuitous that multiple additional ligands could be identified for the HA clearance receptor, because they inhibited HA binding by either binding within the HA site or by occluding or sterically blocking this site when bound to an independent nearby site. After purifying and molecularly cloning the rat and human receptors [35,36,37], the Weigel group discovered an independent Hep binding site [13,29]. This was definitively demonstrated using recombinant cells expressing a HARE mutant lacking the HA-binding Link domain [14]. Although these cells were unable to bind and take up HA, Hep was still bound and rapidly internalized. Harris and Weigel [14] used the 190HARE, rather than full-length Stab2 (315HARE), to assess the binding interactions among eight ligands, the most used in one study. These data enabled development of a scheme for the overlapping ligand interactions associated with the HA and Hep binding sites, based on the abilities of each ligand to compete with other ligands for binding to the 190HARE.

Two multi-ligand competition studies [8,14] provide most of our current knowledge regarding the organization and relationships among Stab2 binding regions for all the soluble ligands (Figure 1A). Also shown are the HARE and Stab2 F1–F7 regions that enable cell engulfment by binding to αMβ2 or αvβ5 integrin on lymphocytes [11,16] and the E1–E4 regions that enable cell engulfment by binding to PS patches on apoptotic cells [27]. The number of ligands identified to bind within the C-terminal half of the Stab2 protein has grown steadily. Two binding sites recognize GAGs; one site, corresponding to the Link domain, binds HA, CS-A, CS-C and CS-D and a second site binds Hep. Although DS and CS-E substantially block Hep binding and do not bind stably to the HA site, they both also partially inhibit HA binding (likely via steric hindrance). Similarly, AcLDL does not block HA binding, although it partially inhibits Hep binding; DS partially blocks AcLDL binding as well. Although HA binding is not inhibited by DS, DS binding is partially blocked by HA [14]. The latter result may reflect steric hindrance of DS binding by much larger HA, hindering access to an independent but proximal binding site. Therefore, the three binding sites for AcLDL, Hep and HA must be functionally and thus spatially organized near each other, likely in tandem, and clearly within the C-terminal half of Stab2 (Figure 1B).

Melkko et al. [8] found that PINP or PIIINP binding is not inhibited by HA but is partially inhibited by AcLDL, fA or pIA; thus, the binding sites for these five ligands are likely on the N-terminal side of the Hep binding site (Figure 1A). This study, performed prior to the cloning era, was only confirmed to show bone fide binding to Stab2 based on the subsequent results of Tamura et al. [10] and Harris and Weigel [14]; both studies showed that independently cloned recombinant receptor binds to AcLDL. Tamura et al. [10] found that AcLDL inhibits binding of ^125^I-AGE-albumin and Harris and Weigel [14] showed that ^125^I-AcLDL binding is partially inhibited by DS, Hep or DxS. These two latter studies, therefore, support a conclusion that the AcLDL binding site is N-terminal to the Hep binding site. Although labeled PINP and PIIINP have not yet been used to show a direct interaction with Stab2, they likely bind to a fourth distinct binding region, since binding of both ligands is partially inhibited by AcLDL. The AGE product binding area is most probably within the Hep and AcLDL binding site region. Finally, pASO binding to recombinant Stab2 is not inhibited by HA but is partially blocked by Hep [17], indicating that this ligand class binds in the general region that mediates binding to Hep, DS, DxS and AcLDL (Figure 1A). The HARE and Stab2 regions that bind bacteria, the plaque-targeting peptide and VWFactor•FactorVIII complexes have not yet been identified.

Based on the reported competition patterns among ligands, we can deduce the overall functional relationships and organization of the binding sites for 16 molecular ligands within the C-terminal half (HARE) portion of Stab2 (Figure 1A): 11 anionic polymers and five proteins. Since PS binds to the E3 and E4 regions [27] and αMβ2 and αvβ5 integrins bind to the F5–F7 regions [11,16], the C-terminal 190HARE region of 315HARE likely participates in the binding and uptake of dying lymphocytes and red blood cells targeted by these ligand interactions with full-length receptor on circulating macrophages. The E1–E4 and/or F1–F7 domains also likely mediate the binding and phagocytosis of Gram–Negative and Gram–Positive bacteria. An important point is that the 16 molecular ligand binding sites have only been identified in the C-terminal half of HARE/Stab2, not the N-terminal half (Figure 1B).

In fact, no binding activity has been reported to be solely within the N-terminal half of HARE/Stab2. Only three studies have examined ligand binding to both 190HARE and 315HARE. Harris et al. [13] found that the soluble purified ectodomains of the 190HARE and 315HARE isoforms specifically bind Hep equally well with a K_d_ of 17.2 ± 4.9 and 23.4 ± 5.3 nM, respectively. In a related study, Harris and Weigel [14] found that HA and Hep bound similarly to the ectodomains of 190HARE and 315HARE. Miller et al. [17] showed that several pASO compounds bind to purified ectodomains of 190HARE or 315HARE and can be internalized by cell expressing either half-length or full-length receptor. Except for these three ligands, there are no other studies on whether the other 13 soluble ligands with binding sites within the 190HARE (Figure 1) are able to bind to full-length 315HARE and half-length 190HARE with similar kinetics, affinities and specificities.

## 7. Cellular Distributions, Pathways and Processing of Hyaluronan and Receptors

My group began studying the liver HA Clearance Receptor in the mid-1980s. We were studying coated pit mediated endocytosis of the asialoglycoprotein receptor in isolated rat hepatocytes and began studying the new HA receptor in the co-isolated SECs. Raja et al. [38] showed in 1988 that rat liver SECs contain a large pool of intracellular HA receptors (75% of the total 500,000 receptors) with the same affinity as surface receptors; K_d_ = 58 nM. In 1989 McGary et al. [33] demonstrated that the HA receptor is a constitutively recycling receptor, with all receptors (surface and intracellular) able to internalize HA. At least 65% of intracellular HA receptors become occupied with HA within 30 min, confirming that the large pool of intracellular receptors functions during continuous endocytosis. A steady-state rate of ligand uptake and degradation is attained in 3–4 h and cultured SECs can sustain this rate of ligand uptake and degradation until no more intact ligand remains. Recycling receptors are generally targeted to coated pits for rapid ligand uptake. McGary et al. showed that HA endocytosis was 90% inhibited by hyperosmolarity [33], which is consistent with uptake mediated by a clathrin coated pit pathway. This was supported by Smedsrod et al. [39], who showed in 1988 that colloidal gold particles coated with HA or CS-A exclusively localize in SEC surface coated pits. Seven years later Hansen et al. [40] confirmed that Stab1 and Stab2 are independently targeted to clathrin coat pits, as expected.

Like other constitutively recycling receptor systems (e.g., the asialoglycoprotein and LDL receptors) that continuously move into clathrin coated pits, with or without bound ligand, HARE and Stab2 are internalized, shunted through multiple endosomal compartments and recycled back to the cell surface [41]. If present, bound ligand is dissociated from receptor in endosomes of lower pH and free ligand is shunted to lysosomes for degradation. The internalized unoccupied receptors then continue along their recycling pathway to be delivered back to the surface, ready to bind other ligands if present and bring them into coated pits in this continuous conveyor belt-like process.

## 8. Functional Molecular Cloning of the Hyaluronan Receptor for Endocytosis

During the 1980s and 1990s, several groups attempted, unsuccessfully, to purify the rat SEC HA receptor. In 1996, Yannariello-Brown et al. [42] developed a ligand blot binding assay, similar technically to Western blotting, to detect HA-binding proteins using an ^125^I-HA derivative. After nonreducing SDS-PAGE of rat SEC membrane proteins, blots treated with ^125^I-HA identified two HA-binding bands (175 and 300 kDa) that were competed >95% with unlabeled HA; these two proteins were found in SECs, but not in hepatocytes. Active HA receptor could thus be monitored during multi-step purification and this allowed Zhou et al. [43] in 1999 and 2000 [44] to purify the rat SEC 175 kDa protein by continuous flow preparative SDS-PAGE and to generate a panel of mouse monoclonal Abs (mAb). One of these, mAb-30, enabled immuno-purification of the two large HA receptors present in liver SECs [43]. Additionally, mAb-174 blocked specific HA binding in blots and cells in vitro and also blocked HA clearance by a perfused liver [45], confirming that the antibody recognizes the native clearance receptor. Receptor expression was very high in liver sinusoids, the venous sinuses of spleen pulp, and the medullary sinuses of lymph nodes.

Based on the above findings, the history of receptor discovery, and that all assays monitored only HA-binding activity for the purification and vetting of reagents, in 1999, Zhou et al. [43] named the protein the *Endocytic HA Receptor*. In 2000, Zhou et al. [44] proposed a variation of this functional name, the *HA Receptor for Endocytosis* (HARE).

## 9. Multiple Groups Clone the Hyaluronan Clearance Receptor in 2002

Zhou et al. [35] molecularly cloned the rat HARE cDNA in 2002, based on peptide sequences obtained from immuno-purified 175HARE. The human 190HARE was then purified and the coding sequence was reported in 2003 by Zhou et al. [36]. Also in 2002, Adachi and Tsujimoto [9] generated cDNA from human umbilical cord endothelial cells and screened transfected cells visually for endocytosis of fluorescent Diiodo-Ac-LDL. After cDNA cloning and based on domains of the recombinant protein the receptor was designated FEEL-1 for: Fasciclin, EGF-like, laminin-type EGF-like, and Link domain-containing scavenger receptor-1. A second paralogous gene sequence FEEL-2 (Stab2) was identified in a database search. Tamura et al. [10] then showed that both FEEL-1 and FEEL-2 were endocytic receptors for AGE-products. The receptor name FEEL has not used subsequently, after the two initial reports.

Also in 2002, Politz et al. [46] used a combination of techniques to identify cDNAs that encoded two large transmembrane proteins with Link domains. The HA clearance receptor was enriched from whole liver extracts by HA-affinity chromatography and peptides from a 270 kDa band in the crude preparation were matched to a genome database, enabling cloning of cDNAs for the mouse and human HA receptors. The authors did not explain its meaning or why they chose the name Stabilin, which seems more appropriate for a structural protein than this very mobile receptor. An unexpected finding, with no supporting data, was that stabilin-1 does not bind HA in a variety of assay formats, despite the presence of the HA-binding Link domain [46]. This very important negative finding was shown directly by Prevo et al. [47] in elegant immunofluorescence studies to assess Stab1 trafficking between the cell surface and endosomes.

Irjala et al. [48] reported cloning a protein, which they acknowledged was Stab1, that they nonetheless renamed CLEVER-1 (common lymphatic endothelial and vascular endothelial Receptor-1). There is only one CLEVER protein in the database and the related Stab2 protein (i.e., CLEVER-2) has not been mentioned. Thus, this 2003 report renamed a known protein, even recognizing by its designation as number-1 that a second member of this receptor family was already known.

So, in a remarkable coincidence 21 years after the discovery of the HA Clearance Receptor, three separate groups reported in 2002 the cloning of two receptor proteins: HARE and Stab2 [9,35,46]. Two groups cloned full-length 315HARE receptor cDNA based on expression selection by AcLDL binding [9] or binding to an HA resin using whole liver extracts [46], which contain many HA-binding proteins [49,50]. The third (Weigel) group cloned the half-length 175HARE rat receptor cDNA based on HA-binding activity and immuno-purification with a highly specific Ab; mAb-30 [35].

Based on results with reagents specific to HARE and Stab2 (e.g., mAbs and mRNA probes), the main tissues expressing these receptors are sinusoidal endothelium in the liver, spleen, lymph nodes and bone marrow [9,35,36,46]. The main expressing cell types are SECs and macrophages, but lower expression is also found in other specialized tissues and cells. Falkowski et al. [51] found HARE and Stab2 protein expression in specialized structures of mouse eyes, hearts, brains, and kidneys, as well as in corneal and lens epithelium, heart valve mesenchymal cells, brain ventricle ependymal cells, and epithelial cells covering renal papillae.

## 10. Stab2 Occurs In Vivo and In Vitro as Two Functional Isoforms Generated by Partial Proteolysis

190HARE is not a splice variant of Stab2. Although multiple Stab2 splice variants have been identified by Hare and Harris [52] in the human liver, spleen and lymph nodes, the 190HARE protein is not a splice variant. Harris et al. [53] showed that human 190HARE is a Stab2 isoform produced by proteolysis of full-length recombinant 315HARE. Since the recombinant gene includes no introns, mRNA splicing is not possible. Nonetheless, the full-length 2551 aa recombinant human protein is specifically cleaved at the N-terminal bond of Ser^1136^ [36,53] to create a 1416 aa 190 kDa type 1 membrane receptor isoform. Since the smaller 1416 aa isoform represents 55.5% of the 2551 aa protein, we refer to it as the C-terminal half of Stab2 (Figure 1B). It is not known if the site where receptor processing occurs is intracellular or at the surface, and the possible functions and fate of the released soluble N-terminal half of the receptor are also unknown.

It is very significant to note that no other group studying Stab2 has examined their systems or assays for the presence and activity of the 190HARE half-receptor isoform. Based on our findings and experience over three decades, the half-receptor is present in all cells producing HARE/Stab2. Proteolytic processing to generate both full- and half-receptors occurs in all cell types tested (e.g., HEK293 and CHO) and all isolated cell lines expressing the recombinant gene [53]. Based on specific ^125^I-HA and mAb binding, proteolytic processing to produce 190HARE and 315HARE membrane isoforms also occurs in vivo in all tissues examined, including the liver, spleen and lymph nodes [42,43,44,45]. The same proteolysis may also occur for Stab1, since major bands at 190 and 300 kDa are immuno-precipitated by mAb against MS-1 antigen; the 300 kDa species was used to obtain the peptides for cDNA cloning [46]. Two large protein isoforms might also have been found during CLEVER-1 cloning [48], but the region <200 kDa was omitted from the immune-blot figures shown. Prevo et al. [47], however, did report two appropriately sized bands for Stabilin-1, and showed that the Stab1 Link domain does not bind HA or other GAGs. Their data support a role for Stab1 as a rapidly recycling scavenger receptor, rather than a cell adhesion or lymphocyte homing receptor.

The 190HARE receptor is twice as abundant as 315HARE in the major clearance tissues. SECs in the three main clearance tissues express more copies of the smaller 190HARE than intact 315HARE. The yield of the two HARE proteins recovered by immuno-purification or as assessed by HA-binding in ligand blot assays shows that the liver, lymph nodes and spleen express about twice as many 190HARE as 315HARE protein molecules, on a molar basis [42,43,44,45]. In these tissues, therefore, SECs cleave about 2/3 of the continuously synthesized 315HARE proteins, thus maintaining a 2:1 molar ratio of the 190:315 HARE isoforms. Such a controlled partial cleavage of a protein cohort may be a more difficult cellular process to control than complete protein cleavage, which is more common.

A major hurdle in studies to assess the ligand binding capability of the full-length protein is that every cell line we tested proteolytically cleaves a fraction of the 315HARE made, creating a mixture of both 190HARE and 315HARE [53]. The complication of intracellular proteolytic processing that creates two functional receptor isoforms in all the studies expressing full-length 315-kDa HARE, FEEL2, or Stab2 means that all published whole cell data reflect the function of two different receptor populations. Until this partial proteolytic cleavage process is eliminated in recombinant cell lines, it will remain problematic to determine whether the binding and internalization abilities and characteristics of the full-length receptor are the same as those of the half-receptor.

## 11. Controlled Proteolysis that Produces Two Receptor Isoforms May Be Necessary for the Very Different Dual Functions of Soluble Ligand Endocytosis and Cell Phagocytosis

There may be strong driving-selection forces for the evolution and continuance of a controlled partial cleavage mechanism that creates two major receptor isoforms in a consistent ratio. A full-length receptor could have evolved so that cell-binding and the mediation of phagocytosis were not affected by the presence of soluble ligands and conversely, soluble ligand uptake might be unaffected by cellular ligands. Theoretically, cell phagocytosis and soluble ligand endocytosis could occur simultaneously with the same cluster of receptor molecules bound to a single cell each also internalizing one or more bound soluble ligands. However, binding to a target cell and mediating its uptake by many thousands of co-ordinated receptors is fundamentally different than soluble ligand uptake mediated by a single or dimeric receptor. It is unlikely that these two diverse uptake processes could be simultaneously mediated by the same receptors, for the reasons noted below.

### 11.1. Cell-Cell Binding Mediated by Full-Length Stab2 May Prevent Simultaneous Soluble Ligand Binding

The binding of one cell to another cell or surface is typically mediated by 10^4^–10^5^ receptors on the capturing cell, that migrate on its surface to form a large patch or cap (~1 µm) at the contact point with a target cell [54]. For macrophage recognition of an apoptotic cell, 315HARE/Stab2 engages with PS (Figure 1) at each of the four E1–E4 domains [12,27,28]. Four receptor–ligand interactions generate much stronger, i.e., higher affinity, binding compared to one interaction. Macrophage recognition of a lymphocyte occurs similarly, via seven F1–F7 domains distributed in four domain clusters. Therefore, full-length receptors have four binding domains for recognition of both PS on apoptotic cells and integrins on lymphocytes (and likely also bacteria). The sites span most of the ectodomain, with ample spacing to accommodate binding to multiple large patches of PS or integrins. The energetics of this binding likely ensure that the long ectodomain is bound to ligand at every site possible. Multivalent associations of tens of thousands of receptors with target cell surface ligands could create a 3-D Velcro-like zone that blocks or sterically occludes soluble ligands from competing for receptor binding sites. Such strong adhesion does not dissociate, except with severe shear force [54]. Thus, it seems unlikely that stable cell–cell binding leading to 315HARE/Stab2-mediated phagocytosis can simultaneously enable the collateral uptake of soluble ligands.

### 11.2. Very High Soluble Ligand Levels Might Impair Full-Length Stabilin2-Mediated Cell Phagocytosis

Despite the above arguments, very high concentrations of multiple soluble circulating ligands (e.g., polymeric GAGs) that bind in or near the E3–E4 and F5–F7 regions might prevent these PS- and integrin-binding domains from interacting with target cells strongly enough to initiate productive phagocytosis. Nonproductive macrophage-cell binding that leads to frustrated phagocytosis can cause inflammation and tissue damage, if lysosomal contents are released externally rather than in phagosomes. Infection, autoimmune and degenerative diseases can accelerate higher biomatrix turnover than normal. Increased circulating levels of released GAGs and collagen propeptides (half the ligands in Figure 1A), could be more likely to interfere with 315HARE/Stab2 driven cell uptake. In unregulated diabetic patients with very high glucose levels, all circulating proteins and cells become more highly glycated and ultimately damaged at above normal frequency. A decreased clearance of cells expressing these AGE ligands could occur if soluble AGE ligand concentrations become too high. Similarly, if no partial cleavage occurs to produce 190HARE, this could drastically decrease ongoing systemic clearance of soluble ligands created by matrix turnover, protein glycation, ethanol metabolism (creating acetaldehyde that can acetylate proteins), etc. Increased soluble ligand levels, in turn, could impair cellular clearance by a full-length receptor. In these above scenarios, a large fraction of available full-length HARE/Stab2 is directed toward cell phagocytosis, at the expense of clearing the circulating soluble ligands. Paradoxically, the higher soluble ligand concentrations might then be able to impair receptor-mediated cell ingestion.

### 11.3. The Mechanisms of Receptor Participation in Endocytosis and Phagocytosis are Very Different

Clathrin-coated pit-mediated endocytosis is very different from phagocytosis. Mechanisms by which a macro-cluster of HARE/Stab2 receptors on a macrophage enable it to bind and ingest a bacterial or apoptotic cell are very different from the mechanisms by which a constitutively recycling HARE/Stab2 receptor continuously moves into and out of a cell mediated by its targeting to clathrin coated pits. The targeting of HARE/Stab2•ligand complexes is mediated by subsets of four cytoplasmic domain endocytic motifs, depending on the ligand bound [55]. During each endocytosis cycle, receptors may bind and internalize soluble ligands that are ultimately delivered to lysosomes after dissociation from a receptor. Receptors bound to macro-ligands can also drive coated pit clustering into macro-patches, but there is no evidence to indicate if either 190HARE or 315HARE is associated preferentially with either a phagocytic or endocytic pathway, or with both.

Although presently unknown, I believe further studies will verify that full-length 315HARE/Stab2 is preferentially associated with the phagocytic pathway, whereas half-length 190HARE/Stab2 is preferentially associated with the endocytic pathway. The hypothesis is that generation of 190HARE half-receptors occurs to create a subset of HARE/Stab2 specialized, perhaps solely, for the function of soluble ligand uptake (removing the body’s daily molecular trash). This would free up and enable full-length receptor to specialize in phagocytosis and the removal of damaged, apoptotic and foreign cells. The evolutionary pathway to create 190HARE from 315HARE was likely driven by selection processes that enabled soluble ligands to be effectively cleared even in the presence of large numbers of cell ligands, whose removal required a large enough fraction of full-length receptors that the extent of small molecule clearance needed for good health or reproduction, etc., was significantly impaired.

The question may not be, “can 315HARE/Stab2 bind soluble ligands in the same way as 190HARE”, but rather, “can soluble ligands be cleared effectively by 315HARE/Stab2 receptor if cell binding and endocytosis dominate its function to the detriment of soluble ligand binding and phagocytosis”.

## 12. Functional Differences between the Hyaluroan Receptor for Endocytosis and Stabilin-2 Should Guide a Nomenclature Change

Most groups studying Stab2 have not recognized the significance of, or examined their recombinant cells for, expression of both 190 kDa and 315 kDa receptor isoforms. Nor do most groups interested in Stab2 appreciate that the 190HARE isoform, rather than the full-length isoform, is responsible for the majority of ligand clearance (Figure 1). In terms of nomenclature, we named the clearance receptor HARE in 2000 [44], two years before the DNA and aa sequences of HARE [35] and Stabilin-2 [46] were reported in 2002. Consequently, both Stab2 and HARE are justifiably official names for the same protein molecularly cloned by two groups in the same year. Nonetheless, using dual names can be confusing, especially since two distinct receptor isoforms are always expressed in the cells tissues responsible for systemic clearance.

Despite not yet knowing exactly where within the protein all 16 ligands bind, a clear trend is apparent (Figure 1). All the single-molecule binding sites are within the C-terminal half of the full-length Stab2 protein, what we designated in 2000 as rat 175HARE or human 190HARE [44]. In contrast, the cell binding sites for PS and integrins are spread out along the whole receptor and, as noted in Section 11, many thousands of full-length proteins are required to enable efficient phagocytosis—e.g., of a target lymphocyte about 10^6^-times larger than a single macrophage HARE/Stab2 receptor bound to it.

Based on all the above considerations, I propose that the current HARE and Stab2 nomenclature that gives dual credit for the molecular cloning be modified to reflect the very significant functional differences between the two receptor isoforms. The designation HARE should now denote the single molecule-binding 190 kDa Stab2 isoform derived by an unknown proteolytic cleavage mechanism. Stab2 should remain the designation of the full-length receptor isoform. This nomenclature change is appropriate for the following reasons.

### 12.1. Two Groups Still Share Credit

Both the Weigel and Goerdt groups would retain their co-naming status for the receptor(s), each with a clear publication path of discovering and cloning a Stab2 isoform. The Goerdt group for first cloning the full-length Stab2 isoform and the Weigel group for first discovering, purifying and cloning the half-length isoform HARE.

### 12.2. Two Names Align with the Presence of Two Receptor Isoforms and May Decrease Confusion

Modified nomenclature would lessen or eliminate confusion generated by having two designations for the receptor and by most investigators not recognizing that there are, in fact, two receptor isoforms. Two different isoform-specific names would emphasize this point and could also simplify discussions.

### 12.3. The Small Isoform was Cloned and Named Based on Hyaluronan Clearance

The small isoform HARE was molecularly cloned based on the first ligand and function identified, the very rapid clearance of HA from blood by liver SECs [2]. In addition, the acronym symbolizes a functional hallmark of recycling receptor-mediated endocytosis: the quickness and speed of a hare (a rabbit relative, genus Lepus, capable of running up to 70 kph).

### 12.4. I Propose Here That The Two Receptor Isoforms Have Evolved Specialized and Different Functions in Order to Mediate Uptake by Two Very Different Processes-Endocytosis versus Phagocytosis

The two isoforms appear to have different specialized functions (Section 11 and Section 13). The small isoform HARE is the major receptor isoform expressed in the liver, spleen and lymph nodes with about twice as many molecules per SEC as full-length Stab2. Thus, the smaller isoform HARE is responsible for the bulk of circulating ligand clearance by SECs in these tissues. Small ligand diffusion access to the smaller 190HARE binding sites on cell surfaces may be easier and less hindered, due to the lack of the large N-terminal half present in the 315HARE protein. Ligand binding kinetics to the smaller 190HARE under flow in vivo might be faster. The large isoform Stab2, is likely the major receptor isoform expressed in macrophages, which circulate in blood and lymph and can also infiltrate tissues. These cells are specialized for Stab2-mediated clearance of macro-scale foreign or apoptotic cells. Full-length Stab2 enables circulating macrophages in the spleen and other tissues to bind, phagocytose and remove large numbers of lymphocytes, red blood cells and tissue cells at daily risk of being damaged, dying (apoptotic) or infected by bacteria or viruses.

### 12.5. Current Evidence Indicates That the Two Isoforms Likely have Distinct and Different Functions

All 16 known single-molecule binding sites are within the 190HARE isoform. Binding sites for these or other molecular ligands have not been reported in only the N-terminal half of the whole Stab2 protein. The HARE designation for this half-receptor isoform recognizes the significance of this functional difference. Of course, a full-length receptor with the same 190HARE aa sequence in its last 1416 aa may be equally competent to bind the 16 soluble ligands in an identical manner, as already shown for HA, Hep and pASO [13,14,17]. However, a major caveat to this assumption is that the identical functionality of the two isoforms could be dependent on their glycosylation profiles being the same. The question is: does proteolytic cleavage occur prior to or after the full processing of all the glycans—e.g., trimming, branching or extension, in both isoforms? If the glycan profiles (i.e., the type of glycans at each site), are different, then the multiple binding activities and functions mediated by the two receptor isoforms could also be different.

Unfortunately, this is a very technically challenging issue to address. For example, human 190HARE contains ~25 kDa of N-linked glycans at 10 of the 17 potential glycosylation sites [56]. Analysis of the various glycans at each site identified from 4–15 different glycans per site and a total of 75 glycan variants at these 10 sites. The possible number of uniquely glycosylated 190HARE species computes to 240 million, a staggering figure; more receptors than a single cell could contain. Although some species are very minor, the point is that deciphering and understanding the importance of glycosylation differences is very difficult and yet glycans can play critical roles in some functions, as we discovered for the 190HARE ectodomain. Within the HA-binding Link domain, Asn^2280^ has 15 different glycans (the greatest structural diversity) and is the only site containing sialylated structures [56]. Membrane-bound HARE lacking Link domain N-glycans mediates rapid HA endocytosis, but purified 190HARE(N2280A) ectodomain shows little or no HA binding; in contrast, HA binding to normal 190HARE ectodomain is high affinity, K_d_ = 5.2 nM. The results indicate that Link domain N-glycans may stabilize interactions that facilitate HA binding to HARE. This example highlights the potential importance of any glycan differences that might be found between the half- and full-length receptor isoforms, and it also illustrates that we cannot assume that the 13 ligand binding sites not yet tested directly with both isoforms will show identical binding characteristics and activities.

I encourage interested readers, who would like to comment on the proposed nomenclature change discussed here to send me your thoughts: at Paul-Weigel@OUHSC.edu.

## 13. A Stress Sensor Model for Hyaluroan Receptor for Endocytosis and Stabilin-2 Functions

In 2012 Weigel et al. [57] proposed a novel *"Tissue-Stress Sensor Hypothesis"* in which the HARE and Stab2 isoforms are proposed to be part of a whole-body sensing system that monitors and responds to the health status of the body’s tissue biomatrices and cells. This system’s baseline activity would be set to maintain tissue steady-state stability and normal ongoing turnover of biomatrices throughout the body. The sensor-system would also detect abnormal cell death and biomatrix turnover and respond to anything that either alters tissue biomatrix composition or turnover or tissue cell viability—e.g., caused by wounds, infection, auto-immune reactions, tumorigenesis or other diseases.

In this model, the sensing system mediated by the two isoforms HARE and Stab2 has both macrophages, as a migratory and circulating surveillance component, and SECs, as a fixed surveillance component in the liver, spleen and lymph nodes. Both cell surveillance components use HARE and Stab2 to co-ordinate and link multiple clearance functions with intracellular responses through signal transduction and gene activation pathways. The downstream stress sensor system responses include the synthesis and secretion of pro- or anti-inflammatory cytokines, such as TGF-β. Harris and Cabral [58], in a companion article in this special issue, summarize the current information about the responses and cell signaling pathways activated when HARE and Stab2 bind and internalize many of the 22 ligands described in this article.

The more abundant receptor may be HARE, relative to Stab2, because it more effectively and rapidly functions to bind and internalize the soluble ligands that circulate continuously within the sinusoidal endothelium of liver, spleen and lymph nodes, the fixed surveillance tissues. Although the steady-state ratio of HARE and Stab2 receptor expression in macrophages is unknown, the prediction based on the functional model proposed here is that Stab2 will be the more abundant isoform. With binding sites that interact with cells throughout the larger Stab2 receptor, it is easier to envision more efficient phagocytosis mediated by Stab2 than could occur with HARE, with half the number of binding sites for ligands on macro-scale ligands. In this special issue, Park and Kim [59] summarize our current understanding of how the full-length Stab1 and Stab2 proteins function as PS receptors to mediate the critical function of phagocytosis.

Future discoveries over the coming decades will undoubtedly clarify, correct, and expand many current ideas and lead to new advances and insights about the function and importance of HARE and Stab2 and other scavenger receptors in human physiology, health and disease.

## Figures and Tables

**Table 1:** Discovery of Ligands for the hyaluronan receptor for endocytosis (HARE) and Stabilin-2.

#	Ligand	Year	Reference
01	Hyaluronan (hyaluronic acid)	1981	[2]
*02*	*Acetylated Low-Density Lipoprotein*	1984	[3]
*03*	*Formylated Albumin*	1984, 1986	[4,5]
04	Chondroitin sulfate-A	1986	[6]
05	Collagen type III propeptide	1988	[7]
06	Collagen type I propeptide	1994	[8]
*07*	*Polyinosinic acid*	1994	[8]
08	Gram–Negative and Gram–Positive bacteria (ligand unknown)	2002	[9]
09	Advanced Glycation End Products	2003	[10]
*10*	*Fucoidan*	2003	[10]
*11*	*Dextran sulfate*	2003	[10]
12	αMβ2 integrin (lymphocytes)	2007	[11]
13	Phosphatidylserine (apoptotic cells)	2008	[12]
14	Heparin	2008	[13]
15	Chondroitin sulfate-C	2008	[14]
16	Chondroitin sulfate-D	2008	[14]
17	Chondroitin sulfate-E	2008	[14]
18	Dermatan sulphate (CS-B)	2008	[14]
19	Stab2-specific peptide (sclerotic plaques)	2011	[15]
20	αvβ5 integrin (lymphocytes)	2012	[16]
*21*	*Phosphorothioate oligonucleotides*	2016	[17]
22	von Willebrand Factor•Factor VIII complex	2018	[18]

The order of discovery for the 22 known ligands listed is indicated sequentially with the relevant publication years and reference numbers. *Italic* font denotes synthetic ligands that are therapeutics (e.g., pASOs) or do not occur naturally in mammals. They likely mimic unknown physiologic molecules—e.g., DxS likely simulates the charge patterns of Hep [26] and AcLDL and fA may mimic natural AGE product ligands.

**Table 2:** HARE and Stabilin-2 ligands.

Number	Ligand Class	Isoform
1	**ANIONIC POLYMERS (11 ligands)**	
	Glycosaminoglycans	
	Chondroitin sulfate-A	HARE
	Chondroitin sulfate-C	HARE
	Chondroitin sulfate-D	HARE
	Chondroitin sulfate-E	HARE
	Dermatan sulfate	HARE
	*Dextran sulfate*	HARE
	*Fucoidan*	HARE
	Heparin	HARE
	Hyaluronan	HARE
	Nucleic Acids	
	*Phosphorothioate oligonucleotide*	HARE
	*Polyinosinic acid*	HARE
2	**PROTEINS (7 ligands)**	
	Modified	
	*Acetylated low density lipoprotein*	HARE
	Advanced glycation end products	HARE
	*Formylated albumin*	HARE
	Cleaved	
	Collagen Type I N-propeptide	HARE
	Collagen Type III N-propeptide	HARE
	VWFactor•FactorVIII complex	HARE
	Peptide	
	Sclerotic plaque-specific targeting	?
3	**CELLS (4 ligands)**	
	Apoptotic cells (phosphatidylserine)	STAB2
	Gram-Negative bacteria	STAB2
	Gram-Positive bacteria	STAB2
	Lymphocytes (αMβ2 or αvβ5 integrin)	STAB2

The 22 receptor ligands are grouped into three categories, two of which are soluble molecular ligands: (1) anionic polysaccharides or synthetic nucleic acids and (2) proteins that are either cleaved or modified at their amine groups; a novel synthetic peptide is included in this group. Group (3) is comprised of cells. Bacterial surface molecules recognized by Stab2 have not yet been identified. The appropriate isoform with a single site or multiple sites for binding each ligand is indicated; either the human C-terminal half-receptor (1416 aa) designated as HARE or the full-length receptor Stab2 (2551 aa). Reasons for the isoform designations are explained in Section 11 and Section 12. *Italic* font denotes nonphysiologic synthetic ligands as in Table 1.

## Abbreviations

aa, amino acid; Ab, antibody; AcLDL, acetylated low density lipoprotein; AGE product, Advanced Glycation End product; CS, chondroitin sulfate; DS, dermatan sulfate; DxS, dextran sulfate; E1-E4, EGF-like repeat domains 1-4; fA, formylated albumin; F1–F7, Fasciclin-1 domains 1-7; Fd, fucoidan; GAG, glycosaminoglycan; HA, hyaluronan, hyaluronic acid; HARE, Hyaluronic Acid Receptor for Endocytosis; 175HARE, 175 kDa HARE, the half-length rat receptor; 190HARE, 190 kDa HARE, the half-length human receptor; 315HARE, 315 kDa HARE, the full-length human receptor; Hep, heparin; mAb, monoclonal antibody; pASO, phosphorothioate antisense oligonucleotide; pIA, polyinosinic acid; PINP, procollagen type I N-terminal propeptide; PIIINP, procollagen type III N-terminal propeptide; PS, phosphatidyserine; Stab, Stabilin; VWF, Von Willebrand Factor.
